# Lethal complication of a rare cardiac tumor

**DOI:** 10.1007/s12024-023-00703-5

**Published:** 2023-09-16

**Authors:** S. Siegel, L. Claus, T. Kamphausen, K. Feld

**Affiliations:** 1 Institut für Rechtsmedizin des Universitätsklinikum Düsseldorf, Moorenstraße 5, 40225 Düsseldorf, Germany; 2 Institut für Pathologie am St. Elisabeth-Krankenhaus Köln, Cologne, Germany; 3 Institut für Rechtsmedizin der Uniklinik Köln, Cologne, Germany; 4 Institut für Rechts- und Verkehrsmedizin des Universitätsklinikum Heidelberg, Heidelberg, Germany

**Keywords:** Cardiac tumor, Autopsy, Sarcoma, Medical malpractice

## Abstract

Cardiac tumors, especially malignant ones, are rare and diagnosis is challenging since symptoms manifest late and are often non-specific. Achieving a histological diagnosis prior to resection is also difficult because biopsies often fail to yield conclusive results. Due to the low frequency, no standard treatment protocol exists and the prognosis is poor. We present a case of a cardiac sarcoma, which was found during an autopsy performed with regard to medical malpractice, because the patient died due to a medical intervention. To report cases like this is important to gain more knowledge about possible complications regarding rare diseases.

## Introduction

Cardiac tumors are very rare [1, 2], the incidence is reported between 0.0017 and 0.28% in autopsy cases [3] and 0.008% in a retrospective analysis of a database [4]. Most cardiac tumors are benign ones, mainly myxoma [2, 3]. Around 25% of cardiac tumors are malignant, with the majority being metastatic [5]. When considering only primary cardiac tumors, the prevalence of malignancy amounts to 9.9% [6]. The most common primary malignant cardiac tumors are sarcomas [5],undifferentiated sarcomas are diagnosed in up to 24% of these cases [1]. During autopsies, cardiac tumors are invariably incidental findings.

Indication for autopsies differs, depending on the formal setting, e.g., clinical vs. forensic autopsy. Also, concerning forensic autopsies, the spectrum of indications varies. With increasing frequency, forensic autopsies are performed to investigate potential medical malpractice [7, 8]. In many cases, autopsy findings can rule out malpractice, on the basis that in criminal law, it is mandatory to state that the patient would have survived a relevant time slot with near certainty [7]. Hence, if during autopsy another potential cause of death arises or significant pre-existing organ damage is identified, confirming medical malpractice with adequate certainty becomes increasingly challenging.

In Germany, it is recommended to state an “undetermined” or “not natural” manner of death when a patient dies due to a medical intervention or on the operating table [9]. We present a case of a cardiac sarcoma and its lethal complication which was found during an autopsy that was performed to investigate potential medical malpractice.

## Case report

### Patient’s history

A 54-year-old male patient was admitted to a hospital due to failure of his transplant kidney and a need of dialysis. During the hospital stay, he developed a persistent cough, so a computed tomography (CT) was performed, which showed pleural and pericardial effusion. An echocardiography demonstrated the hemodynamic significance of these findings. 1.4-L hemorrhagic fluid was aspirated from the pericardial space. Cytology of the fluid revealed numerous granulocytes and occasional atypical cells with uncertain malignancy. Magnetic resonance imaging (MRI) showed an infiltrative growing tumor of the heart, which encased the left anterior descending artery (LAD). A cardiac catheter examination was subsequently conducted, revealing a diagnosis of a triple vessel coronary artery disease (CAD). A biopsy of the tumor showed only necrosis. In spite of the high risk associated with stent implantation for the treatment of CAD which was considered highly risky, both the physicians and the patient himself decided to perform it, given the progressive and severe symptoms. During the subsequent catheterization, LAD was perforated which resulted in a hemorrhagic pericardial tamponade. Despite attempts of aspiration, coronary occlusion, and cardiopulmonary resuscitation (CPR), the patient died.

As he died due to medical intervention, autopsy was performed to investigate any malpractice.

### Autopsy

**Fig. 1:**
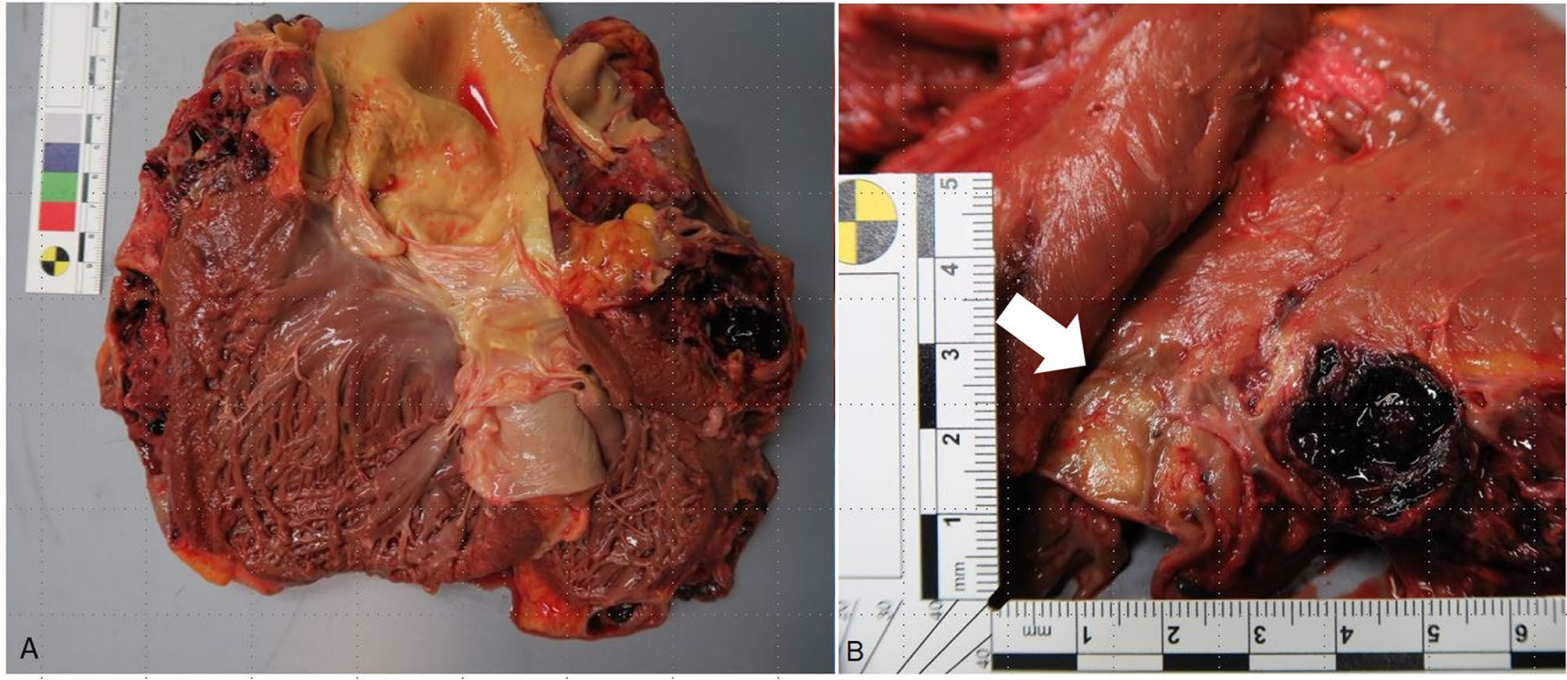
Macroscopic findings.** A** Overview cystic and hemorrhagic tumor. **B** Detailed solid part of the tumor infiltrating the left myocardium (arrow)

Main finding during autopsy was the clinically diagnosed cardiac tumor that completely encased the LAD as described by the clinicians beforehand. The diagnosis of the triple-vessel CAD was also confirmed. Furthermore, the LAD was highly fragile due to sclerosis. The heart weighed 1065 g (incl. tumor) and the tumor size was 10 × 5 × 3 cm. It covered the left ventricle and was predominantly cystic and hemorrhagic. Only a small part of the tumor was solid and seemed to infiltrate the left myocardium (Fig. 1). There was neither another primary tumor nor a metastasis from the heart tumor visible during autopsy.

After the autopsy, no medical malpractice was determined because the tumor and the sclerosis were high-risk factors for the intervention. Moreover, perforation is a potential complication of a catheterization and the physicians reacted to the incident in an appropriate manner.

### Histological findings

**Fig. 2:**
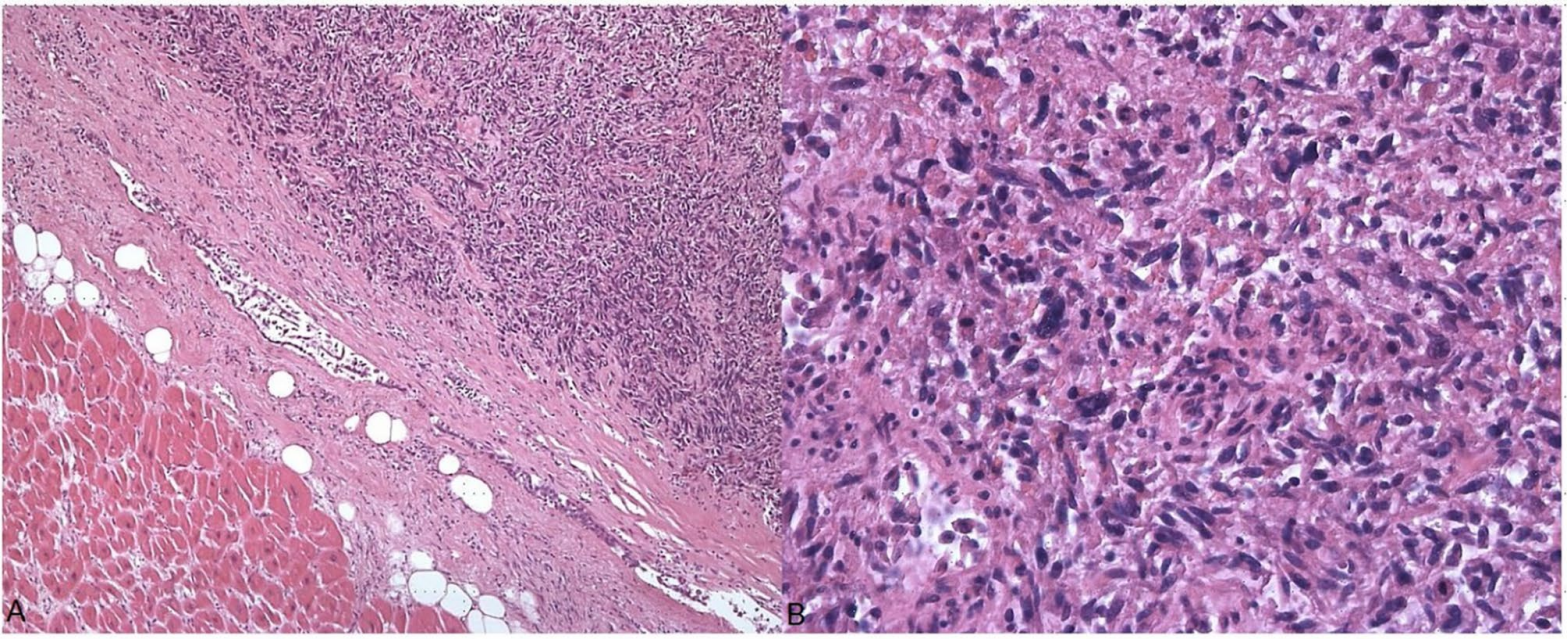
Histological findings.** A** Comparison between normal myocardium (left) and tumor (right). **B** Polymorphic spindle cells

Several samples from the heart and tumor were taken for histological examination during autopsy. Tissues were fixed in 10% formalin solution, processed, and stained with hematoxylin–eosin (HE). Light microscopy showed a tumor growing from the outside into the myocardium. It was predominantly built of densely packed pleomorphic spindle cells (Fig. 2). The nuclei were elongated, pleomorphic, and atypically dark. Some areas appeared epithelioid. There were many small vessels and extensive tumor necrosis (approximately 80%) with signs of old recurring bleedings. The adjacent myocardium presented properly.

Additional immunohistochemical examinations were performed. Vimentin was rated positive. Cytokeratin, desmin, S100, CD31, MOC31, and calretinin were rated negative. SMA and CD34 colored blood vessels in the area of the tumor, but not the tumor cells. Proliferation rate (nuclear Ki-67) was very high, up to 80%.

Based on the histological and the immunohistochemical findings, the tumor was diagnosed as undifferentiated high-grade pleomorphic sarcoma (malignant cardiac fibrous histiocytoma).

## Discussion

Diagnosis of primary cardiac tumors is difficult as symptoms are often non-specific and manifest themselves in particular in heart failure symptoms like dyspnea or cough [5, 10, 11]. Also, symptoms often only occur when the tumor is locally advanced [11]. One main finding of malignant cardiac tumors is pericardial effusion [12].

Clinical diagnostics contains echocardiogram and imaging (CT scan/MRI/positron emission tomography (PET)) [1, 5, 13, 14]. Cytology and biopsy may help to determine the tumor entity but often, similar to this case, do not yield a result [14] and might be risky [13, 15]. Histological and immunochemistry examinations have a higher value during/after resection [13]. Diagnosis of undifferentiated sarcoma is then one of the exclusions [1].

While patients suffering from benign cardiac tumors can often be treated successfully [5, 16], treatment of cardiac sarcomas is challenging, partly due to the absence of a standard treatment protocol owing to their low frequency [10, 17]. Primarily, resection is suggested but the prognosis is poor [2, 10, 11]. The role of heart transplantation in cases of irresectable tumors is unclear [18]. Also, the benefits of (neo)adjuvant chemotherapy remain unclear [11, 17, 18] while radiotherapy might be recommended in irresectable tumors [18]. Mean survival time of patients with malignant cardiac tumors is 7 months to 2 years [5].

In our case, the tumor had grown very big (10 × 5 × 3 cm; heart weight, 1065 g) at the time of diagnosis. The first symptom was a cough. One primary finding was a massive pericardial effusion. The tumor was hemorrhagic which presented an additional risk factor for the cardiac catheterization along with the sclerosis and the encasement of the LAD. Furthermore, it consisted predominantly of necrotic material which plausibly explains why the biopsy did not yield a diagnosis.

Only some case reports and series about malignant cardiac tumors—e.g., Hamidi et al. [11], Chen et al. [19], and Alam et al. [10]—can be found in current literature. Given the low incidence of such tumors and the resulting absence of clinical guidelines, it is important to publish these cases in order to gain a better understanding of the disease and its potential progression. This applies especially for cases of lethal complications to improve clinical deliberations, for example, regarding upcoming medical interventions.

## Key points

1. Primary malignant cardiac tumors are rare, diagnosis is difficult and therapy often poor.
2. Cardiac tumors may go along with higher risks in cases of medical intervention, such as catheterization.
3. Given the low frequency of primary malignant cardiac tumors, it is important to report these cases to improve medical treatment.
4. Autopsies could, on the one hand, help gain a better understanding and on the other hand, they could be performed to rule out medical malpractice in cases of (lethal) complications.
